# Socioeconomic determinants of shift work employment in Canada and the Netherlands: a cross-sectional analysis of data from the Canadian Longitudinal Study on Aging and the Hoorn Study

**DOI:** 10.3389/fpubh.2026.1699140

**Published:** 2026-03-19

**Authors:** Dennis B. Campbell, Tucker Reed, Romy Slebe, Parminder Raina, Femke Rutters, Divya Joshi, Renée de Mutsert, Jean-Pierre Despres, Joris Hoeks, Denis P. Blondin, André Carpentier, Patrick Schrauwen, David J. T. Campbell

**Affiliations:** 1 Department of Medicine, University of Alberta, Edmonton, AB, Canada; 2 Department of Community Health Sciences, Cumming School of Medicine, University of Calgary, Calgary, AB, Canada; 3 Department of Epidemiology and Data Science, Amsterdam UMC, Amsterdam, Netherlands; 4 Health Behaviours and Chronic Diseases, Amsterdam Public Health, Amsterdam, Netherlands; 5 Department of Health Research Methods, Evidence, and Impact, McMaster University, Hamilton, ON, Canada; 6 McMaster Institute for Research on Aging, McMaster University, Hamilton, ON, Canada; 7 Department of Clinical Epidemiology, Leiden University Medical Center, Leiden, Netherlands; 8 Department of Kinesiology, Faculty of Medicine, Université Laval, Sainte-Foy, QC, Canada; 9 VITAM–Centre de Recherche en Santé Durable, Sainte-Foy, QC, Canada; 10 Centre de Recherche de l'Institut Universitaire de Cardiologie et de Pneumologie de Québec, Université Laval, Sainte-Foy, QC, Canada; 11 Department of Nutrition and Movement Sciences, NUTRIM School of Nutrition and Translational Research in Metabolism, Maastricht University, Maastricht, Netherlands; 12 Centre de recherche du Centre hospitalier universitaire de Sherbrooke, Sherbrooke, QC, Canada; 13 Department of Medicine, Division of Neurology, Faculty of Medicine and Health Sciences, Université de Sherbrooke, Sherbrooke, QC, Canada; 14 Department of Medicine, Division of Endocrinology, Faculty of Medicine and Health Sciences, Université de Sherbrooke, Sherbrooke, QC, Canada; 15 Institute for Clinical Diabetology, German Diabetes Center, Leibniz Institute for Diabetes Research at Heinrich Heine University Düsseldorf, Düsseldorf, Germany; 16 Department of Medicine, Cumming School of Medicine, University of Calgary, Calgary, AB, Canada; 17 Department of Cardiac Sciences, Cumming School of Medicine, University of Calgary, Calgary, AB, Canada

**Keywords:** circadian alignment, CLSA, Hoorn Study, shift work, socioeconomic disadvantage

## Abstract

**Background:**

Shift work is known to be a risk factor for metabolic diseases. Although not established within literature, socioeconomic disadvantage may be associated with increased risk of being engaged in these shift work patterns.

**Aim:**

To examine whether socioeconomic factors are associated with the likelihood of engaging in shift work.

**Methods:**

Using data from the Canadian Longitudinal Study on Aging (CLSA), we conducted a cross-sectional analysis of adults aged 45–85 years. We created a Social Disadvantage Score composed of points derived from immigration status, language, education and race, ranging from 0 to 6. Logistic regression models were used to examine the association between social disadvantage and shift work, while adjusting for relevant covariates. We performed similar analyses using data from the Hoorn Study in the Netherlands—focused on the association between educational attainment and shift work.

**Results:**

Of 18,393 adults employed in the CLSA at baseline, 16.6% reported currently working in a shift work scheduled job. Odds of shift work employment increased progressively with Social Disadvantage Score. Individuals with the highest scores (4+) had 3.25 times the odds (95% CI: 2.24–4.72) of being employed in shift work compared to those with a score of 0. Of 709 adults in the Hoorn Study, 11.4% worked in shift work patterns. Lower education was associated with shift work employment (OR 1.74, 95% CI: 1.1–2.9).

**Conclusions:**

Social disadvantage is strongly associated with shift work in Canada, and in the Netherlands, lower education is associated with shift work.

## Introduction

In recent years, evidence of the negative impacts of circadian misalignment between the endogenous circadian system and 24-hour behavioral/environmental cycles on human health has continued to build (1, 2). Circadian rhythms are established by a central biological clock located in the suprachiasmatic nucleus of the hypothalamus with numerous peripheral clocks in tissues throughout the body (3). Ideally, the endogenous internal clocks are synchronized with the environmental rhythm (3), which is comprised of various behavioral “Zeitgebers”—or signals—such as food intake, sleep, light, and physical activity (4).

Unfortunately, all too common in our modern lifestyle, our behaviors are dyssynchronous with our endogenous clock, including prolonged screen time, light at night, late night eating, reductions in sleep, and working long hours (5, 6). These disruptions in circadian rhythms have been implicated in various conditions, particularly in cardiometabolic diseases including hypertension (7), obesity (8), cardiovascular disease (CVD) (9, 10), and type 2 diabetes (7, 11, 12). Engaging in shift work is particularly problematic, as it affects a number of Zeitgebers, including light exposure, sleep timing, and meal timing (6). Poor sleep quantity and quality, known to result from shift work (7), have also been associated with cardiometabolic diseases (13, 14). Circadian misalignment has also been associated with a range of other adverse health outcomes, including cognitive impairment; however, cardiometabolic disease provides the primary biological context motivating the present study (15).

It is also well understood that those facing socioeconomic disadvantage are at highest risk of developing cardiometabolic diseases (16) and also have worse outcomes, compared to counterparts who face fewer socioeconomic disadvantages (17). However, it is unclear if there is an association between socioeconomic disadvantage and shift work, though it is postulated that those who face a greater degree of social disadvantage may be more likely to work in jobs where shift work and long hours are common. This, however, has not been clearly established in the literature to date.

The objective of this study was to determine whether socioeconomic factors such as education, race/ethnicity, and immigration status are associated with an increased odds of being engaged in shift work, both in Canada and in the Netherlands. Understanding this association will help fill the gap in understanding some of the potential mechanisms underlying the increased risk of cardiometabolic diseases faced by those with socioeconomic disadvantages.

## Materials and methods

### Design and setting

We performed paired cross-sectional analyses in Canada and the Netherlands. In Canada, we used self-reported baseline data from the Canadian Longitudinal Study on Aging (CLSA) from 2011–2015. The study was approved by the Hamilton Integrated Research Ethics Board at McMaster University (Project ID: 14106). In the Netherlands, we used data from Wave two of the Hoorn study, collected in 2013–2015. The Ethics Committee of the VU University Medical Center approved the Hoorn Study (2012/222), and written informed consent was obtained from all participants.

### Study population

The Canadian Longitudinal Study on Aging (CLSA) is a national, longitudinal research platform including 51,338 participants aged 45–85 years at baseline from all Canadian provinces. This age range was intended by the investigators to be able to capture middle aged experiences that influence aging as well as older adults who have reached advanced age while living in the community. Details on the study design have been described elsewhere (18).

The sampling frames for the study included: [1] Participants in the Canadian Community Health Survey who provided their contact information and consent to be contacted for the CLSA; [2] Information packages were mailed to randomly chosen individuals from provincial healthcare databases on behalf of the CLSA; and [3] Random digit dialing of landlines based on area codes, if an eligible individual within contacted households were willing, they provided their individual contact information for further interviews and assessments (19). All participants in the study were selected using stratified random sampling from the community-dwelling population. To be eligible for the study, participants had to be physically and cognitively able to participate on their own and communicate in English or French. Members of the armed forces, individuals living in Canada's three territories or institutions such as long-term care facilities, and persons living on federal First Nation reserves and other First Nations settlements in the provinces were not eligible to participate in the study. Participants were recruited in the tracking cohort (*n* = 21,241) and the comprehensive cohort (*n* = 30,097). Tracking cohort participants were randomly selected from Canadian provinces and completed interviews by phone. Participants in the comprehensive cohort were randomly selected from within 25–50 km distance of 11 data collection sites, which are located in seven Canadian provinces. In addition to being interviewed in-person, comprehensive cohort participants completed in-depth physical assessments and provided blood and urine samples (18).

For the present study, we excluded those who were not working at the time of baseline survey administration or those partially retired (*n* = 31,343), as the nature of the survey questions surrounding employment schedules most directly applied to current workers rather than those who had retired or taken leave. We also excluded those who report a current casual or seasonal position (*n* = 755), as we did not have sufficient detail on their actual day-to-day work schedule. Lastly, we excluded those with missing data (*n* = 847) in variables related to our Social Disadvantage Score (described below).

The Hoorn Study is a population-based cohort started in 1989, representative of the general Dutch population. To date, two waves of data have been collected, one in 1989 and one in 2006. In wave two, a total of 6,180 inhabitants of the city of Hoorn, aged 40–65 years and able to provide consent were randomly selected from the municipal registry. Of these, 45.4% agreed to participate in the cohort. Between January 2013 and December 2015, 1,734 participants agreed to participate in the follow-up. A complete overview of the study design is provided elsewhere (20). The primary objective of the Hoorn Study is to identify biological, behavioral, and environmental factors that influence the development and progression of type 2 diabetes and its complications. Consequently, the dataset contains limited socioeconomic variables, and proxy indicators of socioeconomic position were used to approximate these factors. For this current analysis, we only used data from the 2013–2015 measurements, as questions on shift work were only assessed at that time point. Of the 1,734 individuals who participated in the follow-up, we excluded participants who were not working at the time of administration (*n* = 982), had missing data on education level (*n* = 4) or had missing data on included covariates (*n* = 39).

### Variables

*Outcome:* in the CLSA, shift work was defined based on response to the following question: “Which of the following best describes your working schedule currently?”. Response options included “Daytime schedule or shift”, “evening shift”, “night shift”, “rotating shift changing periodically from days to evenings or nights”, and “other”. In our analysis, we dichotomized these options into “daytime schedule/shift” and “all others” (which were classified as shift workers).

In the Hoorn Study, shift work was defined based on the response to the survey questions: “Do you currently have a paid job” and “Do you work in shift work or at a nighttime schedule?”. In our analysis, we only included participants that were working at the time of administration and dichotomized this group into “daytime workers” and “shift/nighttime workers”.

*Exposure:* in the CLSA, self-reported indicators of socioeconomic disadvantage included race (categorized as: white or racialized population), immigration status (Canadian-born, immigrated >10 years ago, and immigrated < 10 years ago), first language (categorized as non-minority or minority first language, based on province of residence [non-French in Quebec, and non-English outside of Quebec]), and educational attainment (high education: university degree or higher; medium education: high-school degree or some post-secondary; low education: no high school degree). We chose these indicators as they are included in the Statistics Canada's dimensions of multiple deprivation (21). To understand the compounding and incremental effect of these various socioeconomic contributors, we created a bespoke Social Disadvantage Score ranging from 0–6 with points given for increasing levels of social disadvantage. That said, we merged scores of 4+ into one category as few individuals had scores of 5 and 6 (see Box 1). We did not include income in our Social Disadvantage Score as it potentially represents a downstream consequence of several other included factors and could therefore disproportionately drive the observed association between the overall score and shift work.

Box 1 Parameters of the social disadvantage score.

**Table:** 

**Category**	**Points given**
Immigration status	0—Canadian-born
	1—Immigrated >10 years ago
	2—Immigrated < 10 years ago
Language	0—Non-minority language
	1—Minority language
Education	0—High education (university degree)
	1—Medium education (high-school degree or some post-secondary)
	2—Low education (no high-school degree)
Race	0—White
	1—Visible minority or Indigenous ancestry

In the Hoorn Study, educational attainment was used as a proxy for socioeconomic disadvantage because other related socioeconomic variables were not available. Education levels were dichotomized into two groups: low and high. Low education included no formal education, primary or elementary school, lower vocational education, or intermediate general secondary education. High education included intermediate vocational education, higher general or pre-university education, higher professional education, or university-level education. This dichotomization was applied to maintain adequate cell sizes across categories and is consistent with previous analyses of the Hoorn Study (22).

*Covariates*: there are several other factors which are associated with both socioeconomic disadvantage and shift work and may therefore act as confounding factors. These were controlled for in our regression analysis. These include age (grouped as 45–54, 55–64, 65+ years), sex (self-reported male *vs*. female), and location of residence (urban *vs*. rural). In the CLSA, urban/rural status was determined by Statistics Canada's Postal Code Conversion File (23). In the Hoorn Study, this was based on the total number of addresses *per* km^2^ and dichotomized into urban in case of one thousand or more addresses per km^2^ and into rural in case of fewer than one thousand addresses per km^2^ (24).

### Statistical analyses

For both CLSA and Hoorn analyses, we calculated descriptive statistics stratified by current employment schedule (shift/night work *vs*. regular daytime schedule). The CLSA data were weighted in order to account for sample misrepresentation due to unequal sampling probabilities, frame coverage error and non-response, and to improve the precision of estimates. Inflation weights, derived from weights given by Statistics Canada, were used to balance the sample to be more representative of the Canadian population (19).

We performed a multivariable binary logistic regression to calculate odds ratios of shift work associated with socioeconomic disadvantage. Fully adjusted models included the potential confounders of interest: age, sex, and urban/rural as covariates. With the CLSA data, we used frequency weights which were derived from normalized analytic weights. These analytic weights were rescaled inflation weights summed to the sample size within each province, so that their mean value was 1 within each province. In the CLSA data, we first modeled each indicator individually, followed by modeling our Social Disadvantage Score with the same adjustment variables. Analyses were done using Stata, version 17 (StataCorp) for CLSA data and SPSS, version 28 for Hoorn Study data.

## Results

### Study cohort and respondent characteristics

A total of 18,393 individuals who reported being currently employed were included in the analysis from the CLSA cohort. Average age of CLSA participants was 54.7 years (SD: 6.4) with 47.9% being female. The majority (15,347 participants, 83.4%) reported currently working in a regular daytime schedule leaving 3,046 participants (16.6%) working in some form of a shift work schedule. Each of the covariates showed similar distribution when stratified by work schedule (Table 1).

**Table 1:** Descriptive characteristics of participants in the CLSA at baseline, stratified by work schedule (*n* = 18,393).

**Variable**		**Total (*n =* 18,393)**	**Daytime workers (*n =* 15,347)**	**Shift workers (*n =* 3,046)**
Age group	45–54 years	10,448 (56.8%)	8,670 (56.9%)	1,778 (56.4%)
	55–64 years	6,669 (36.3%)	5,507 (36.1%)	1,162 (36.9%)
	65–85 years	1,276 (6.9%)	1,063 (7.0%)	213 (6.8%)
Sex	Male	9,576 (52.1%)	7,980 (52.4%)	1,596 (50.6%)
	Female	8,817 (47.9%)	7,260 (47.6%)	1,557 (49.4%)
Residence	Urban	15,085 (82.0%)	12,652 (83.0%)	2,433 (77.1%)
	Rural	2,450 (13.3%)	1,944 (12.8%)	506 (16.1%)
	Missing	858 (4.7%)	644 (4.2%)	214 (6.8%)
Education	High education (university degree)	15,001 (81.6%)	12,636 (82.9%)	2,365 (75.0%)
	Medium education (high-school degree or some post-secondary)	2,844 (15.5%)	2,229 (14.6%)	624 (19.8%)
	Low education (no high-school degree)	548 (3.0%)	384 (2.5%)	164 (5.2%)
Immigration status	Canadian-born	15,624 (85.0%)	12,933 (84.9%)	2,691 (85.4%)
	Immigrated >10 years ago	2,564 (13.9%)	2,166 (14.2%)	398 (12.6%)
	Immigrated < 10 years ago	205 (1.1%)	141 (0.9%)	64 (2.0%)
Language	Non-minority language	15,639 (85.0%)	12,937 (84.9%)	2,702 (85.7%)
	Minority language	2,754 (15.0%)	2,303 (15.1%)	451 (14.3%)
Race	White	17,217 (93.6%)	14,306 (93.9%)	2,911 (92.3%)
	Visible minority or indigenous ancestry	1,176 (6.4%)	934 (6.1%)	242 (7.7%)

*n* represents the number of individuals in the sample. Percentages reflect weighted prevalence estimates.

A total of 709 individuals were included in the analysis involving the Hoorn cohort. The average age in the included cohort was 56.8 years (SD: 5.12 years), and 50.5% were males. There were 628 (88.6%) participants currently working in a regular daytime schedule, leaving 81 (11.4%) working in shift work schedules (Table 2).

**Table 2:** The Hoorn Study cohort characteristics, stratified by work schedule (*n* = 709).

**Variable**		**Total (*n =* 709)**	**Daytime workers (*n =* 628)**	**Shift workers (*n =* 81)**
Age group	45–54 years	251 (35.4%)	214 (34.1%)	37 (45.7%)
	55–64 years	408 (57.5%)	365 (58.1%)	43 (53.1%)
	65–74 years	50 (7.1%)	49 (7.8%)	1 (1.2%)
Sex	Male	358 (50.5%)	320 (51.0%)	38 (46.9%)
	Female	351 (49.5%)	308 (49.0%)	43 (53.1%)
Residence	Urban	545 (76.9%)	481 (76.6%)	64 (79.0%)
	Rural	164 (23.1%)	147 (23.4%)	17 (21.0%)
Education level	Low	188 (26.5%)	160 (25.5%)	28 (34.6%)
	High	521 (73.5%)	468 (74.5%)	53 (65.4%)

*n* represents the number of individuals in the sample. Percentages reflect weighted prevalence estimates.

### Socioeconomic factors and shift work

**Figure 1:**
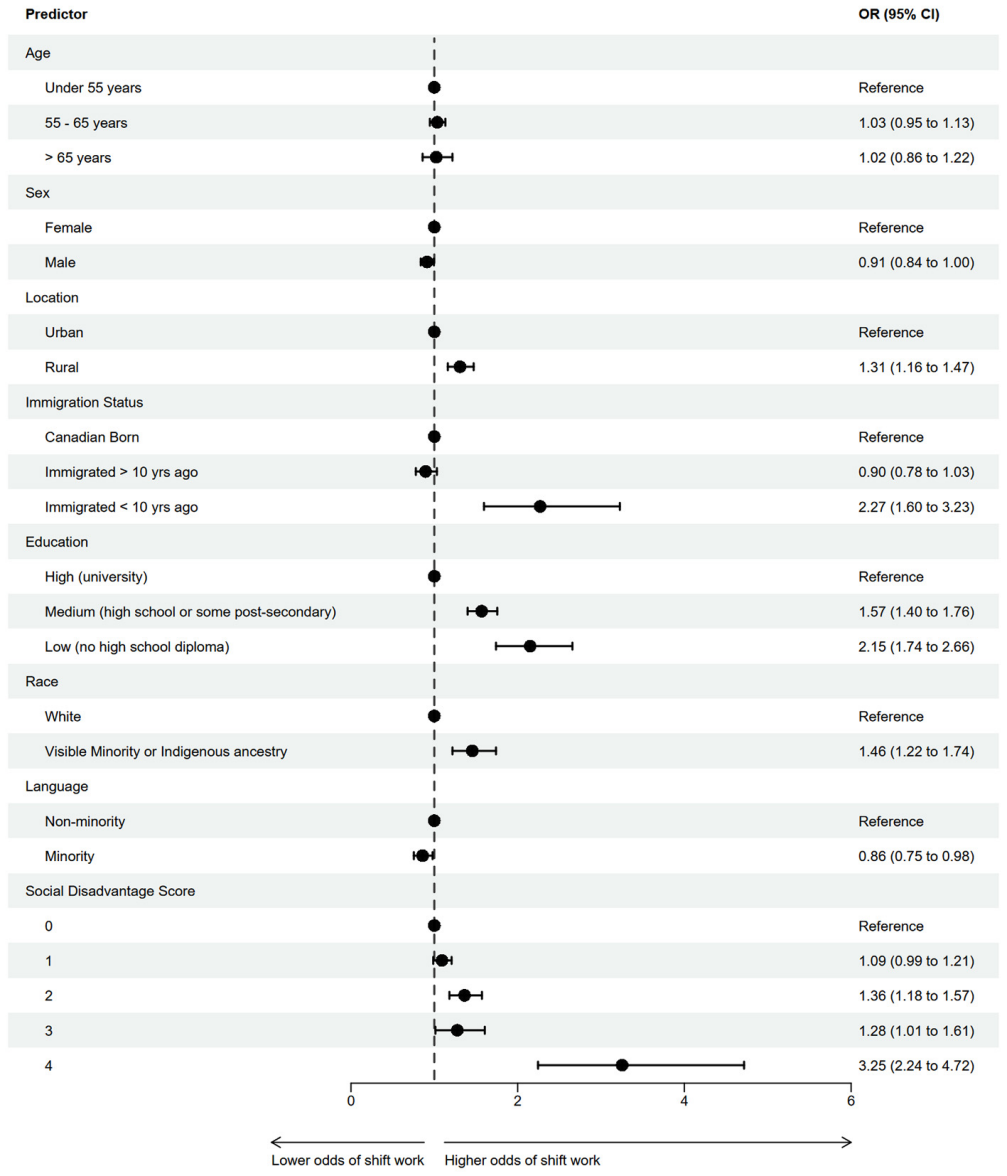
Forest plot showing adjusted odds ratios for shirt work employment associated with each component of the Social Disadvantage Score and selected demographic characteristics from a multivariable model (*n* = 18,393).

When included individually in separate adjusted models in the CLSA, education, race, language, and immigration within the previous 10 years were all associated with shift work while immigration beyond 10 years and language were not individually associated with shift work (Figure 1). Of these indicators, immigration within 10 years showed the strongest association with those individuals having 2.27 times the odds (95% CI: 1.60–3.23) of working in a shift work scheduled job, compared to Canadian-born workers. This association was followed by education, where those with the lowest level of education (no high-school diploma) had 2.15 times the odds (95% CI: 1.74–2.66) of those who had the highest educational attainment (university degree); and race, where individuals from visible minority populations had 1.46 times the odds of shift work (95% CI: 1.22–1.74), compared to white individuals. Interestingly, individuals whose first language was a minority within their province of residence had lower odds of engaging in shift work (OR: 0.86; 95% CI: 0.75–0.98) compared to those whose first language was not a minority.

With respect to the Social Disadvantage Score, the total proportion of respondents with a score of 0 was 11,052 (60.1%), 1 was 4,814 (26.2%), 2 was 1,731 (9.4%), 3 was 627 (3.4%), and 4+ was 169 (0.9%). Modeling our Social Disadvantage Score showed increasing odds of engaging in shift work with higher Social Disadvantage Score. Individuals with a Social Disadvantage Score of 4+ had 3.25 (95% CI: 2.24–4.72) times higher odds of engaging in shift work compared to the reference group (score of 0; Figure 1).

Using the Hoorn data, the crude model assessing the association between education level and shift work found that those with low education had an odds ratio of 1.55 (95% CI: 0.95–2.53), compared to those with high education. After adjustment for age, sex and urban *vs*. rural residency, the association was significant, with odds of shift work for individuals with a low education being 1.74 (95% CI: 1.1–2.9) times higher, compared to participants with a higher education.

## Discussion

Of the almost 20,000 working adults in the CLSA cohort, about 17% reported currently working in a shift work scheduled job. In the CLSA, odds of shift work employment increased progressively with Social Disadvantage Score, with individuals in the group with the most social disadvantage (score of 4+) having 3.25-times higher odds of performing shift work compared to those who did not experience social disadvantage (score of 0). Those with lower levels of education, and those from visible minority populations were more likely to engage in shift work, while the strongest predictor was immigration within 10 years. In the Dutch population, 11.4% of individuals were working a shift work scheduled job. When adjusting for covariates, those with low levels of education had 1.75-fold higher odds of engaging in shift work, when compared to those with the highest levels of education—a very similar finding to the results in the Canadian cohort.

Our study findings are consistent with those from other studies that have looked at risk factors for shift work. One study from the US found that immigrants from African/Caribbean countries (32.5%) and those with Hispanic ethnicity (31.9%) had twice the likelihood of working night shifts compared to non-Hispanic white individuals (15.6%) (25). Interestingly, the rate of shift work in these minority groups was considerably higher than the rates observed in our cohorts where 20.6% of racialized minorities worked in shifts, whereas our studies had a relatively comparable rate of white individuals working in shifts (16.9%). In Singapore, ethnicity was also noted to be a key determinant of being occupied in a shift work schedule (26). In Canada, specifically, one systematic review demonstrated that education, and subjective socioeconomic status have been linked with disturbed sleep patterns, some of which is due to shift work (27). In the Netherlands, one other study has shown a similar association between education and shift work, with shift work rates of 26.5% and 16.6% for low education and high education, respectively (28).

One's work environment and colleagues can have a significant impact on health behaviors and practices. For example, it has been shown that people are more likely to engage in healthy behaviors when encouraged to do so by their fellow employees (29). To mitigate the increased risks of shift work patterns and the resulting social disparity, employers could create policies to mitigate the risks of shift work on employees' long-term health. There is already work being done on this front as a number of studies have evaluated the impact of workplace initiatives that encourage healthy behaviors, physical activity programs (30), occupational health guidelines targeted at weight gain prevention among employees (31), and employer-sponsored financial incentives for healthy behaviors (32). A European systematic review found many of these programs and initiatives to be effective at improving participant dietary outcomes (33) while another systematic review targeted specifically at shift workers found evidence for physical activity interventions in weight loss and improved physical fitness (34). In addition to promoting healthy behaviors in the workplace, there are specific strategies to address the unique challenges of shift work and mitigate its negative effects. Evidence suggests that limiting consecutive night shifts, ensuring adequate recovery time between shifts, and reducing overall shift durations can help reduce long-term health issues (35).

Given the increasing knowledge we have of the impact of circadian misalignment on metabolic dysfunction, the increased exposure to shift work seen in these populations may account for some degree of this increased risk of conditions such as type 2 diabetes, and CVD.

One strength of our study was the large population-based cohorts in two different countries. The CLSA included a nationally generalizable sample of participants with a wealth of information on socioeconomic disadvantage indicators and demographic characteristics. Though the findings from the CLSA may be hard to generalize outside of a Canadian context, the use of Hoorn study data, though limited by the number of socioeconomic variables available, helps illustrate similar results in a different setting. A principal weakness of this study was the older age of the cohort. With a minimum age of 45 years in the CLSA, we did not have access to much of the younger working population where people may be even more likely to work in shift-type schedules. Therefore, our findings may not be generalizable to a younger cohort, who may have different patterns of employment than those of older generations. However, cardiometabolic diseases have higher incidence and prevalence in middle-aged and older individuals. Finally, our study only looked at the association between social disadvantage and shift work but was not powered to examine for associations with health outcomes such as dysglycemia, obesity, or cardiovascular events. In future studies, with appropriate data and sample size, one could examine if shift work plays a mediating role between socioeconomic disadvantage and specific clinical outcomes like those mentioned above.

In conclusion, we have shown that many dimensions of social disadvantage are associated with shift work exposure in Canada, while in the Netherlands specifically, lower education was associated with shift work. These work-related disparities which pre-dispose individuals to circadian misalignment may partially explain the increased rate of cardiometabolic diseases seen in groups that face socioeconomic disadvantage. The potential mediating effect of shift work in these associations remains to be tested in future studies.

## Data Availability

The raw data supporting the conclusions of this article will be made available by the authors, without undue reservation.
